# Unravelling the complete mitochondrial genomes of *Thrips tabaci* Lindeman and *Thrips parvispinus* Karny (Thysanoptera: Thripidae) and their phylogenetic implications

**DOI:** 10.3389/finsc.2025.1536160

**Published:** 2025-02-28

**Authors:** P.S. Soumia, Dhananjay V. Shirsat, Vadivelu Karuppaiah, Pratap A. Divekar, Vijay Mahajan

**Affiliations:** ^1^ Crop Protection Section, ICAR-Directorate of Onion and Garlic Research, Pune, Maharashtra, India; ^2^ Division of Crop Protection, ICAR- Indian Institute of Vegetable Research, Varanasi, Uttar Pradesh, India

**Keywords:** invasive pest, mitochondrial genome, phylogeny, *Thrips parvispinus*, *Thrips tabaci*

## Abstract

Onion (*Allium cepa* Linnaeus) is an important vegetable crop valued for its nutritional properties and economics worldwide. Onion cultivation faces serious threats from pests and diseases, particularly onion thrips (*Thrips tabaci*), which cause substantial yield losses. Recently, Black thrips (*Thrips parvispinus*), an invasive key pest of chili, have been reported to cause severe damage in onion crop and is likely to devastate the onion cultivation in near future. Therefore, this study was conducted to address the knowledge gap concerning the genetic basis and evolutionary history of *T. tabaci* and *T. parvispinus* through sequencing of their mitochondrial genomes. *T. tabaci* and *T. parvispinus* were collected from different locations in Maharashtra, India, and reared in the laboratory. The mitochondrial genomes of *T. tabaci* and *T. parvispinus* were sequenced to a length of 15,277 and 15,285 bp, respectively. Both genomes exhibited similar gene organization with regard to thirteen protein-coding genes and two *rRNA* genes. *T. tabaci* contained 19 *tRNA* genes whereas *T. parvispinus* contained 18 *tRNA* genes. The evolutionary positions of *T. tabaci* and *T. parvispinus* within the Thysanoptera order were elucidated through phylogenetic analysis of the mitogenomes of 15 thrips species. These findings provide crucial insights into the genetic makeup and evolutionary dynamics of both the thrips species, thereby aiding the development of novel and sustainable pest management strategies to mitigate their impacts on crops in the changing climate scenario.

## Introduction

Onion (*Allium cepa* L.) is widely acknowledged as an important vegetable crop worldwide, extensively grown and consumed for its culinary and nutritional value, and plays a vital role in the economies of many countries (1). Onion cultivation is particularly widespread in India, which is one of the world’s leading producer and exporter (2). However, onion production faces numerous challenges due to the prevalence of various pests and diseases, which lead to substantial yield losses (3). Globally, onion growers are concerned about onion thrips, *Thrips tabaci* Lindeman (Thysanoptera: Thripidae) (4, 5). In India, this pest is considered of national significance, causing 30% to 40% yield loss in onion crops (6, 7). Thrips infestation not only weakens the plants but also makes them susceptible to secondary infections and diseases, which exacerbates economic impacts on farmers (8, 9). These pests are known vectors for various plant viruses, including the iris yellow spot virus (8, 10), and they also transmit diseases like Stemphylium leaf blight and bacterial leaf blight (3, 11–13). Virus-vector relationship have been well documented in the case of thrips; however, their interaction with fungal pathogens remains largely unexplored (Saini et al., 2024). Moreover, thrips usually exhibit genetic heterogeneity, which might be due to the spatial variation in insecticide efficacy (14). Based on mitochondrial DNA sequences, *T. tabaci* has been classified into three biotypes: one associated with tobacco and two associated with leek (L1 and L2) (15). Also, due to the anticipated increase in temperature, the lifecycle of *T. tabaci* is likely to shorten, leading to multiple generations within a single crop season (16). Currently, onion growers rely on chemical pesticides to manage these pests, but it often seems futile due to their overlapping generations, concealed feeding behavior, and growing insecticide resistance (17, 18).

Similarly, *Thrips parvispinus* (Southeast Asian thrips or black thrips), are known to infest a variety of host plants, including vegetables, ornamentals, and field crops. Recently, *T. parvispinus* has been found infesting onion crops (19), which could pose a substantial threat in the near future (20). The inclusion of *T. parvispinus* in the list of onion pests reveals a potential gap in our understanding of thrips species interactions and their impact on onions. A comprehensive understanding of the biology, genetics, and evolutionary relationships of thrips is required to tackle these pests in onion and to devise appropriate management strategies. Recent advancements in molecular biology have enabled researchers to explore the genetic makeup of various organisms, providing insight into their evolutionary histories and ultimately helping in devising novel pest management strategies.

The increasing interest in mitochondrial genomes for phylogenetic studies has led to a surge in published mitogenome sequences, particularly amongst insects. Although the complete mitochondrial genomes of several thrips species, such as *Thrips imagines* (21), *Frankliniella occidentalis* (22), *Frankliniella intonsa* (23), *Scirtothrips dorsalis* (24), *Anaphothrips obscurus* (25), *Thrips palmi* (26), *Dendrothrips minowai* (27), *Thrips hawaiiensis* (28), *Thrips parvispinus* (29) and *Aptinothrips stylifer* (30), have been sequenced, comprehensive research specifically focusing on onion thrips is unexplored. Insect mitogenomes are small, circular, and consist of 37 genes: 13 protein-coding genes (PCGs), two ribosomal RNA genes (rRNAs), and 22 transfer RNA genes (tRNAs), along with a large A+T-rich control region (CR) that regulates transcription and replication (31, 32). Their maternal inheritance, conserved gene content, and rapid evolutionary rate make mitogenomes valuable molecular markers for evolutionary research (33). The significant variation in mitochondrial genome organization observed within the subfamily Thripinae is highly unusual and contrasts with patterns seen in most other animals. The reasons behind the rapid evolution of mitochondrial genomes in Thripinae, as well as the evolutionary dynamics of mitochondrial genomes in other thrips, remain to be explored (25). In this context, the present study on the complete mitogenome of *T. tabaci* and its phylogenetic implications is of great significance. Furthermore, comparative studies of different thrips mitogenomes will help in elucidating the evolutionary patterns and population dynamics within the Thysanoptera order. Therefore, the study aims to address the knowledge gap by presenting the complete mitochondrial genome sequences of *T. tabaci* and *T. parvispinus*. This will offer valuable insights into their evolutionary relationships, population structure, and genetic diversity, ultimately aiding in devising an effective pest management strategy.

## Materials and methods

### Sample collection

Adults of onion thrips were collected from onion plants and initially reared in the laboratory on French beans at the ICAR-Directorate of Onion and Garlic Research (ICAR-DOGR) in Pune, Maharashtra, India (latitude: 18.84°N, longitude: 73.88°E, 616.29 meters above sea level). After completing their life cycle, adults emerged from individual eggs, were collected and used for further analysis. Meanwhile, Black thrips from chili plants in a farmer’s field (latitude: 18.87°N, longitude: 74.05°E, elevation: 667.84 meters) were collected and preserved in 99% ethanol for further analysis. The species identity of these specimens was confirmed through DNA barcoding of the *COX-1* gene, and their sequence information has been submitted in the NCBI GeneBank database with accession numbers PP980527 and PP982736 for *T. tabaci* and *T. parvispinus* respectively.

### Sample preparation and DNA isolation

Single adults of *T. tabaci* and *T. parvispinus* were macerated in liquid nitrogen, and total genomic DNA was extracted using the DNeasy Blood and Tissue Kit (QIAGEN, Germany). The integrity of isolated total DNA was visualized on 1% agarose gel and further quantified using a nanodrop (Bio-Rad, Hercules, California, USA). Mitochondrial DNA was then synthesized from the total DNA using the REPLI-g Mitochondrial DNA Kit (QIAGEN, Germany).

### Sequence assembly, annotation, and analysis

The genome library was constructed with the QIASeq FX DNA kit (QIAGEN, Germany), and sequencing was performed on an Illumina NextSeq 2000 platform using 300-cycle paired-end chemistry, generating primary FASTQ data. These FASTQ files were assessed for total bases, read counts, GC%, Q30, and uncertain base percentages. Reads of high quality were obtained by eliminating adaptor contamination, ambiguous reads, and junk sequences using the fastp tool (v0.12.4) (34). Subsequently, BWA MEM (v0.7.17) was used to align the cleaned reads to the reference sequence (35). Protein-coding and RNA genes were identified from the consensus sequence with SAM tools’ mpileup (36). A *de novo* assembly using a de Bruijn graph approach was performed to construct longer DNA contigs, and the mitogenome was assembled using the MEGAHIT tool (37) as part of the MitoZ package (38). The quality of assembly was evaluated using the Quast tool (39).

Gene annotation for the mitogenome was conducted with the Prokka annotation tool (40) on the Proksee web server (https://proksee.ca/) (41), producing a circular genome map, GC concentration, and GC skew. For tRNA gene structure prediction and mitogenome assessment, MITOS2 (42) at the Galaxy Europe Web Server (https://usegalaxy.eu/) was used.

The relative synonymous codon usage (RSCU), codon usage, and base composition (A+T contents) of the PCGs were analyzed using MEGA Software (v11.0.13) (43). The GC skewness was computed using the formula GC skew = (G-C)/(G+C); whereas the formula AT skew = (A-T)/(A+T) was used for determining the AT skewness (44). Intergenic spacers and gene overlaps were manually determined. The complete mitochondrial genomes of *T. tabaci* and *T. parvispinus* were submitted to the NCBI genome database under accession numbers PQ197393 and PQ197392, respectively.

### Phylogenetic analysis

The evolutionary studies related to *T. tabaci* and *T. parvispinus* with the metagenome of 14 other thrips species were analyzed using the NCBI retrieved sequence information. The damsel bug, *Alloeorhynchus bakeri*, was used as an outgroup species. The nucleotide sequences of 13 protein coding genes of 16 thrips species and one outgroup species were aligned individually using MAFFT 7, the Database of Aligned Structure Homologue (DASH) was utilized to incorporate homologous structures based on amino acid codons (45). Followed by removal of the ambiguously aligned sites, the aligned amino acid sequences were then converted to the nucleic acid sequence. For phylogenetic analysis the sequences of 13 PCGs were concatenated in following order: nad5, nad4, nad4L, nad6, cox1, nad3, cox2, cox3, atp6, atp8, nad1, nad2 and cytB in a single sequence of each species. All the 17 sequences were aligned using CLustalW tool (46). The phylogenetic tree was constructed using the Maximum Likelihood method in MEGA 11 software (43), employing the general time reversible model with gamma distribution (GTR+G) and bootstrap values from 500 iterations.

## Results

### Structure and composition of mitogenome

**Figure 1:**
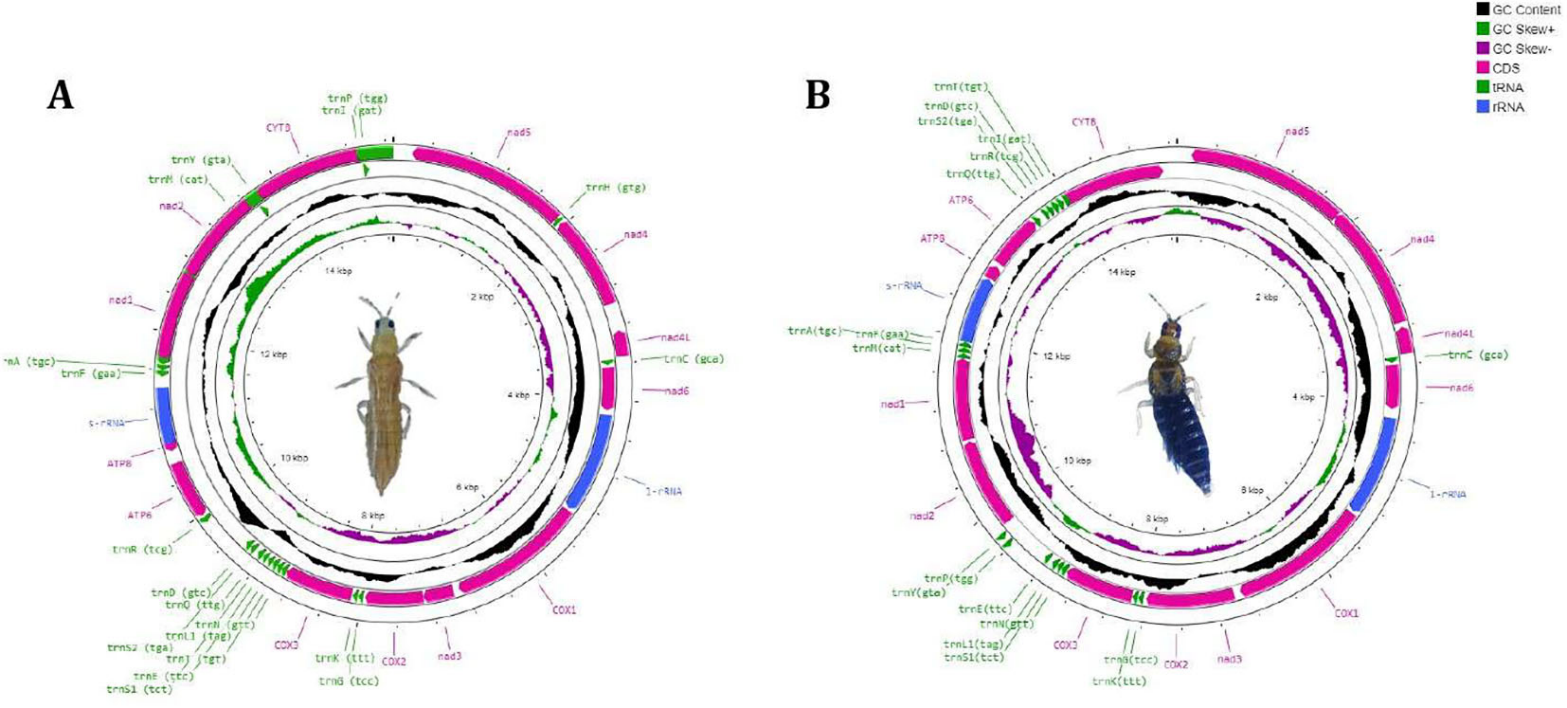
Circular genome map of **(A)** *T. tabaci* and **(B)** *T. parvispinus* showing mitogenome sequence features.

The circular genome of *T. tabaci* is 15,277 bp long (**Figure 1A**), comprising 34 sequence elements (such as 13 PCGs, 19 tRNAs, and 2 rRNA-coding genes). Strand localization analysis revealed 19 genes located on the H-strand (+), whereas 15 genes on the L-strand (–) (**Table 1**). Gene length in the *T. tabaci* mitochondrion was 400 bp on average, with minimum and maximum lengths of 57 bp (trnS1) and 1695 bp (nad5), respectively. Base compositions of the complete mitochondrial genome of *T. tabaci* were 41.3%, 34.8%, 12%, and 11.9% for A, T, G, and C nucleotides. The 13 PCGs and two rRNAs had AT contents ranging from 69.80% to 82.50%, whereas the overall mitogenome had 76.10%. In contrast, the GC content varied from 17.50% to 30.20% for the 13 PCGs and 2 rRNAs, whereas the overall mitogenome had 23.90%.

**Table 1:** Summary of *Thrips tabaci* mitochondrial genome.

Gene Name	Full Name and Function	Gene Type	Position		Length (bp)	Strand	Intergenic Space	Codon		Anti-codon
			Start	End				Start	Stop	
nad5	NADH dehydrogenase subunit 5	PCG	205	1899	1695	–	-12	att	tag	–
trnH	Transfer RNA for Histidine	tRNA	1888	1948	61	–	10	–	–	gtg
nad4	NADH dehydrogenase subunit 4	PCG	1959	2966	1008	–	292	att	t	–
nad4L	NADH dehydrogenase subunit 4L	PCG	3259	3531	273	–	26	atg	taa	–
trnC	Transfer RNA for Cysteine	tRNA	3558	3617	60	+	31	–	–	gca
nad6	NADH dehydrogenase subunit 6	PCG	3649	4122	474	+	51	att	taa	–
rrnL	16S ribosomal RNA	rRNA	4174	5325	1152	+	22	–	–	–
COX1	cytochrome c oxidase subunit I	PCG	5348	6889	1542	+	65	ata	taa	–
nad3	NADH dehydrogenase subunit 3	PCG	6955	7305	351	+	-1	att	taa	–
COX2	cytochrome c oxidase subunit II	PCG	7305	7964	660	+	11	ata	taa	–
trnG	Transfer RNA for Glycine	tRNA	7976	8037	62	+	3	–	–	tcc
trnK	Transfer RNA for Lysine	tRNA	8041	8102	62	+	10	–	–	ttt
COX3	cytochrome c oxidase subunit III	PCG	8113	8895	783	+	0	ata	taa	–
trnN	Transfer RNA for Asparagine	tRNA	8896	8959	64	+	-3	–	–	gtt
trnT	Transfer RNA for Threonine	tRNA	8957	9019	63	+	-1	–	–	tgt
trnL1	Transfer RNA for Leucine	tRNA	9019	9082	64	+	2	–	–	tag
trnE	Transfer RNA for Glutamic acid	tRNA	9085	9147	63	+	8	–	–	ttc
trnS1	Transfer RNA for Serine	tRNA	9156	9212	57	+	6	–	–	tct
trnQ	Transfer RNA for Glutamine	tRNA	9219	9286	68	+	37	–	–	ttg
trnS2	Transfer RNA for Serine	tRNA	9324	9387	64	+	0	–	–	tga
trnD	Transfer RNA for Aspertic acid	tRNA	9388	9451	64	+	451	–	–	gtc
trnR	Transfer RNA for Arginine	tRNA	9903	9968	66	–	18	–	–	tcg
ATP6	ATP synthase F0 subunit 6	PCG	9987	10607	621	–	50	ata	taa	–
ATP8	ATP synthase F0 subunit 8	PCG	10658	10860	203	–	-54	att	t	–
rrnS	12S ribosomal RNA	rRNA	10807	11413	607	–	116	–	–	–
trnF	Transfer RNA for Phenylalanine	tRNA	11530	11594	65	–	-1	–	–	gaa
trnA	Transfer RNA for Alanine	tRNA	11594	11656	63	–	2	–	–	tgc
trnM	Transfer RNA for Methionine	tRNA	11659	11721	63	–	-4	–	–	cat
nad1	NADH dehydrogenase subunit 2	PCG	11718	12641	924	–	6	ata	taa	–
nad2	NADH dehydrogenase subunit 1	PCG	12648	13628	981	–	73	att	taa	–
trnY	Transfer RNA for Tyrosine	tRNA	13702	13765	64	+	-3	–	–	gta
CYTB	cytochrome b	PCG	13763	14908	1146	–	-29	ata	tag	–
trnI	Transfer RNA for Isoleucine	tRNA	14880	14943	64	–	2	–	–	gat
trnP	Transfer RNA for Proline	tRNA	14946	15008	63	+	268	–	–	tgg

The AT skewness for the 13 PCGs ranged from 0.426 to 0.033, higher than that of the rRNA genes, rrnS (-0.193) and rrnL (0.180) (**Table 2**). The 1560 bp of intergenic nucleotides were spread across 23 locations, with individual spacer lengths ranging from 2 to 451 bp. The longest intergenic spacer (451 bp) was found between the trnD and trnR genes. There were overlaps between nine genes, whose lengths varied from 1 to 54 bp. The atp8 and rrnS genes had the lengthiest overlap, 54 bp, as shown in **Table 1** and **Figure 1A**.

**Table 2:** Nucleotide composition of *Thrips tabaci* mitogenome.

Gene	T	C	A	G	Total	GC%	AT%	GC Skew	AT Skew
nad5	53.2	10.9	25.2	10.7	1695	21.60	78.40	-0.009	-0.357
nad4	51.8	10.8	24.4	13	1008	23.80	76.20	0.092	-0.360
nad4l	56.4	8.8	22.7	12.1	273	20.90	79.10	0.158	-0.426
nad6	44.7	9.9	37.8	7.6	474	17.50	82.50	-0.131	-0.084
cox1	37.5	15.9	32.3	14.3	1542	30.20	69.80	-0.053	-0.074
nad3	38.7	12.9	38.7	9.7	351	22.60	77.40	-0.142	0.000
cox2	35.6	16.2	36.2	12	660	28.20	71.80	-0.149	0.008
cox3	39.4	16.4	32.4	11.8	783	28.20	71.80	-0.163	-0.097
atp6	37.7	14.8	36.2	11.3	621	26.10	73.90	-0.134	-0.020
atp8	41.3	14.3	32.5	11.9	126	26.20	73.80	-0.092	-0.119
nad1	37.3	15.4	35.5	11.8	924	27.20	72.80	-0.132	-0.025
nad2	39.1	12	41.8	7.1	981	19.10	80.90	-0.257	0.033
cob	40.6	14.7	32.7	12	1146	26.70	73.30	-0.101	-0.108
rrnS	45.8	12.2	31	11	607	23.20	76.80	-0.052	-0.193
rrnL	32.5	9.8	46.8	10.9	1152	20.70	79.30	0.053	0.180

Likewise, the circular mitogenome of *T. parvispinus* is 15,285 bp, comprising 13 PCGs, 18 tRNA-coding genes, and 2 rRNA-coding genes, 28 of which are located on the H-strand (+) the remaining 5 on the L-strand (–). Gene length in the *T. parvispinus* mitochondrion was 428 bp on average, with minimum and maximum lengths of 61 bp (trnS1) and 1695 bp (nad5), respectively (**Figure 1B**, **Table 3**). Our result was in accordance with Pakrashi et al. (29), revealing a mitogenome length of 15,067 bp. The mitogenome of *T. parvispinus* had base compositions of 43.3% A, 30% T, 10% G, and 11.7% C, with an overall AT content of 73.3% and GC content of 21.7%. The AT content across the 13 PCGs and 2 rRNAs ranged from 72.8% to 84%, while the GC content varied from 17% to 27.2%. AT skewness was higher in the PCGs (0.064 to 0.410) compared to the rRNAs (0.161 for rrnS and 0.187 for rrnL) (**Table 4**). We identified 1,039 bp of intergenic nucleotides across 24 locations, with the longest spacer (347 bp) between the trnE and trnP genes. Seven genes had overlaps, with the largest (35 bp) between the COX2 and ND4 genes (**Table 3**, **Figure 1B**).

**Table 3:** Summary of *Thrips parvispinus* mitochondrial genome.

Gene Name	Full Name and Function	Gene Type	Position		Length (bp)	Strand	Intergenic Space	Codon		Anti-codon
			Start	End				Start	Stop	
nad5	NADH dehydrogenase subunit 5	PCG	146	1840	1695	–	-5	att	taa	–
nad4	NADH dehydrogenase subunit 4	PCG	1836	3157	1322	–	47	ata	t	–
nad4L	NADH dehydrogenase subunit 4L	PCG	3205	3486	282	–	25	atg	taa	–
trnC	Transfer RNA for Cysteine	tRNA	3512	3574	63	+	18	–	–	gca
nad6	NADH dehydrogenase subunit 6	PCG	3593	4090	498	+	100	att	taa	–
rrnL	16S ribosomal RNA	rRNA	4191	5338	1148	+	20	–	–	–
cox1	cytochrome c oxidase subunit I	PCG	5359	6930	1572	+	71	att	taa	–
nad3	NADH dehydrogenase subunit 3	PCG	7002	7343	342	+	-35	att	taa	–
cox2	cytochrome c oxidase subunit II	PCG	7309	8001	693	+	23	ata	taa	–
trnG	Transfer RNA for Glycine	tRNA	8025	8087	63	+	3	–	–	tcc
trnK	Transfer RNA for Lysine	tRNA	8091	8154	64	+	2	–	–	ttt
cox3	cytochrome c oxidase subunit III	PCG	8157	8945	789	+	4	ttg	taa	–
trnN	Transfer RNA for Asparagine	tRNA	8950	9016	67	+	-4	–	–	gtt
trnS1	Transfer RNA for Serine	tRNA	9013	9073	61	+	0	–	–	tct
trnL1	Transfer RNA for Leucine	tRNA	9074	9138	65	+	42	–	–	tag
trnE	Transfer RNA for Glutamic acid	tRNA	9181	9246	66	+	347	–	–	ttc
trnP	Transfer RNA for Proline	tRNA	9594	9659	66	–	34	–	–	tgg
trnY	Transfer RNA for Tyrosine	tRNA	9694	9757	64	–	57	–	–	gta
nad2	NADH dehydrogenase subunit 2	PCG	9815	10825	1011	+	17	att	taa	–
nad1	NADH dehydrogenase subunit 1	PCG	10843	11772	930	+	-4	att	taa	–
trnM	Transfer RNA for Methionine	tRNA	11769	11833	65	+	1	–	–	cat
trnA	Transfer RNA for	tRNA	11835	11896	62	+	-1	–	–	tgc
trnF	Transfer RNA for Phenylalanine	tRNA	11896	11960	65	+	-2	–	–	gaa
rrnS	12S ribosomal RNA	rRNA	11959	12711	753	+	8	–	–	–
atp8	ATP synthase F0 subunit 8	PCG	12720	12879	160	+	41	att	t	–
atp6	ATP synthase F0 subunit 6	PCG	12921	13535	615	+	5	att	taa	–
trnQ	Transfer RNA for Glutamine	tRNA	13541	13609	69	+	59	–	–	ttg
trnS2	Transfer RNA for Serine	tRNA	13669	13733	65	+	3	–	–	tga
trnD	Transfer RNA for Aspertic acid	tRNA	13737	13801	65	+	1	–	–	gtc
trnR	Transfer RNA for Arginine	tRNA	13803	13870	68	+	0	–	–	tcg
trnT	Transfer RNA for Threonine	tRNA	13871	13935	65	+	10	–	–	tgt
trnI	Transfer RNA for Isoleucine	tRNA	13946	14012	67	+	-32	–	–	gat
cytB	cytochrome b	PCG	13981	15126	1146	+	101	ata	taa	–

**Table 4:** Nucleotide composition of *Thrips parvispinus* mitochondrial genome.

Gene	Length	T	C	A	G	GC%	AT%	GC Skew	AT Skew
**nad5**	1695	54.65	8.40	26.35	10.60	19.00	81.00	0.116	-0.349
**nad4**	1322	55.45	7.60	25.25	11.70	19.30	80.70	0.212	-0.374
**nad4l**	282	59.20	6.75	24.80	9.25	16.00	84.00	0.156	-0.410
**nad6**	498	40.40	9.60	42.00	8.00	17.60	82.40	-0.091	0.019
**cox1**	1572	36.40	14.20	36.40	13.00	27.20	72.80	-0.044	0.000
**nad3**	342	38.60	11.70	40.90	8.80	20.50	79.50	-0.141	0.029
**cox2**	693	34.50	14.30	39.20	12.00	26.30	73.70	-0.087	0.064
**cox3**	789	36.75	15.50	36.45	11.30	26.80	73.20	-0.157	-0.004
**nad2**	1011	40.50	11.60	42.50	5.40	17.00	83.00	-0.365	0.024
**nad1**	930	40.75	12.85	35.40	11.00	23.85	76.15	-0.078	-0.070
**atp8**	160	38.10	11.30	42.50	8.10	19.40	80.60	-0.165	0.055
**atp6**	615	39.20	13.30	37.10	10.40	23.70	76.30	-0.122	-0.028
**cob**	1146	40.00	13.75	35.10	11.15	24.90	75.10	-0.104	-0.065
**rrnL**	1148	32.35	9.10	47.25	11.30	20.40	79.60	0.108	0.187
**rrnS**	753	33.30	10.40	46.10	10.20	20.60	79.40	-0.010	0.161

### Protein-coding genes and codon usage bias

The mitochondrial genomes of *T. tabaci* and *T. parvispinus* exhibit distinct characteristics in their PCGs. The total lengths of the 13 PCGs were 10,584 bp for T*. tabaci* and 11,055 bp for *T. parvispinus*. In *T. tabaci*, the coding sequences ranged from 126 bp (atp8) to 1,542 bp (cox1), with genes such as cox1, cox2, cox3, nad3, and nad6 located on the H-strand, while others like nad1, nad2, nad4, nad4L, nad5, atp6, atp8, and cytB were on the L-strand. Conversely, in *T. parvispinus*, the coding sequences ranged from 160 bp (atp8) to 1,695 bp (nad5), with most genes situated on the H-strand, except nad4, nad4L, and nad5, which were on the L-strand.

Three start codons (ATT, ATA, and ATG) were identified in the *T. tabaci* mitogenome. ATT was adopted by genes like nad2, nad3, nad4, nad5, nad6, and atp8, whereas genes like cox1, cox2, cox3, atp6, nad2, and CytB used ATA. However, ATG was used as the start codon by the nad4L gene. In contrast, *T. parvispinus* utilized four start codons, with ATT being the most prevalent across 8 genes (cox1, atp6, atp8, nad1, nad2, nad3, nad5, and nad6), while 3 genes (nad4, cox2, and cytB) used ATA as their start codon. The ATG and TTG start codons are used by nad4L and cox3, respectively. The ATN start codon is the most common starting codon in most thrips and other insect species (26, 47–49). For stop codons, *T. tabaci* primarily used TAA, but nad5 and cytB used TAG, and nad4 and atp8 used an incomplete stop codon “T”. Similarly, *T. parvispinus* mostly used TAA, with nad4 and atp8 also adopting the incomplete “T”. Incomplete termination codons are common in other thrips species (26, 47) and insect mitochondrial genomes and are presumed to be restored through post-transcriptional polyadenylation (48, 49).

**Figure 2:**
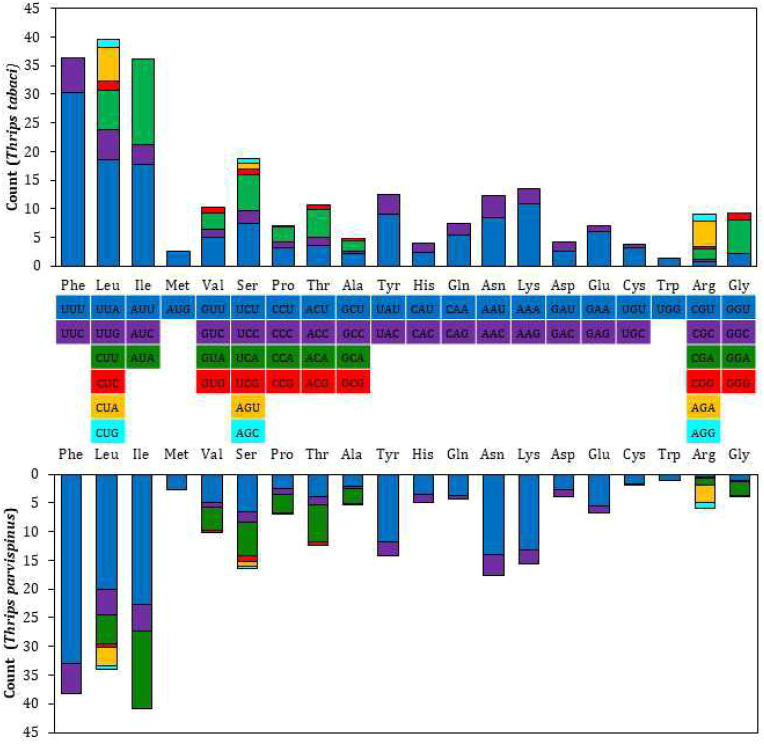
Relative synonymous codon use (RSCU) of 13 PCG’s of the *T. tabaci* and *T. parvispinus*.

The RSCU and amino acid usage in the PCGs of *T. tabaci* and *T. parvispinus* are summarized in **Figure 2** and **Table 5**. In *T. tabaci* mitogenome, leucine, phenylalanine, isoleucine, serine, lysine, tyrosine, and valine were the most frequent amino acids, whereas tryptophan, methionine, and cyanine were the rarest. In general, UUU, followed by UUA, AUU, AUA, and AAA, was the most frequently used codon. However, in the *T. parvispinus* mitogenome, isoleucine, phenylalanine, leucine, asparagine, serine, lysine, and tyrosine were the most frequently used amino acids, whereas tryptophan, cyanine, and methionine were the rarest, with UUU, followed by AUU, UUA, AAU, and AUA being the most frequently used codons (**Table 5**).

**Table 5:** Amino acid usage in the 13 PCG’s of the *T. tabaci* and *T. parvispinus*.

Codon	*T. tabaci*	*T. parvispinus*	Codon	*T. tabaci*	*T. parvispinus*	Codon	*T. tabaci*	*T. parvispinus*	Codon	*T. tabaci*	*T. parvispinus*
UUU(F)	30.3	33	UCU(S)	7.5	6.6	UAU(Y)	9	11.8	UGU(C)	3.1	2.6
UUC(F)	6.3	5.2	UCC(S)	2.2	1.8	UAC(Y)	3.5	2.5	UGC(C)	0.6	0.6
UUA(L)	18.5	20.1	UCA(S)	6.2	5.8	UAA(*)	7	5.7	UGA(*)	4.6	4.1
UUG(L)	5.3	4.4	UCG(S)	1.1	1.1	UAG(*)	2.3	1.9	UGG(W)	1.3	1.2
CUU(L)	6.9	5.1	CCU(P)	3.2	2.6	CAU(H)	2.3	3.5	CGU(R)	0.8	1
CUC(L)	1.7	0.5	CCC(P)	0.9	1	CAC(H)	1.7	1.4	CGC(R)	0.3	0.2
CUA(L)	5.7	3.2	CCA(P)	2.8	3.2	CAA(Q)	5.5	3.8	CGA(R)	1.8	2.2
CUG(L)	1.5	0.7	CCG(P)	0.2	0.2	CAG(Q)	2	0.6	CGG(R)	0.5	0.2
AUU(I)	17.8	22.6	ACU(T)	3.5	3.9	AAU(N)	8.5	14.1	AGU(S)	1	2.2
AUC(I)	3.4	4.8	ACC(T)	1.5	1.5	AAC(N)	3.7	3.6	AGC(S)	0.7	1.4
AUA(I)	14.9	13.5	ACA(T)	4.8	6.3	AAA(K)	10.8	13.2	AGA(R)	4.4	5.8
AUG(M)	2.5	2.8	ACG(T)	0.9	0.7	AAG(K)	2.6	2.5	AGG(R)	1.3	1.9
GUU(V)	5.1	5	GCU(A)	2.1	2.2	GAU(D)	2.6	2.8	GGU(G)	2.1	2.5
GUC(V)	1.4	0.8	GCC(A)	0.4	0.3	GAC(D)	1.5	1.1	GGC(G)	0.1	0.2
GUA(V)	2.8	3.9	GCA(A)	1.8	2.6	GAA(E)	6	5.5	GGA(G)	5.9	5.5
GUG(V)	1	0.4	GCG(A)	0.5	0.2	GAG(E)	1	1.2	GGG(G)	1.2	0.5

The symbol * represent the “Stop codons”.

### Transfer RNAs and ribosomal RNAs

**Figure 3:**
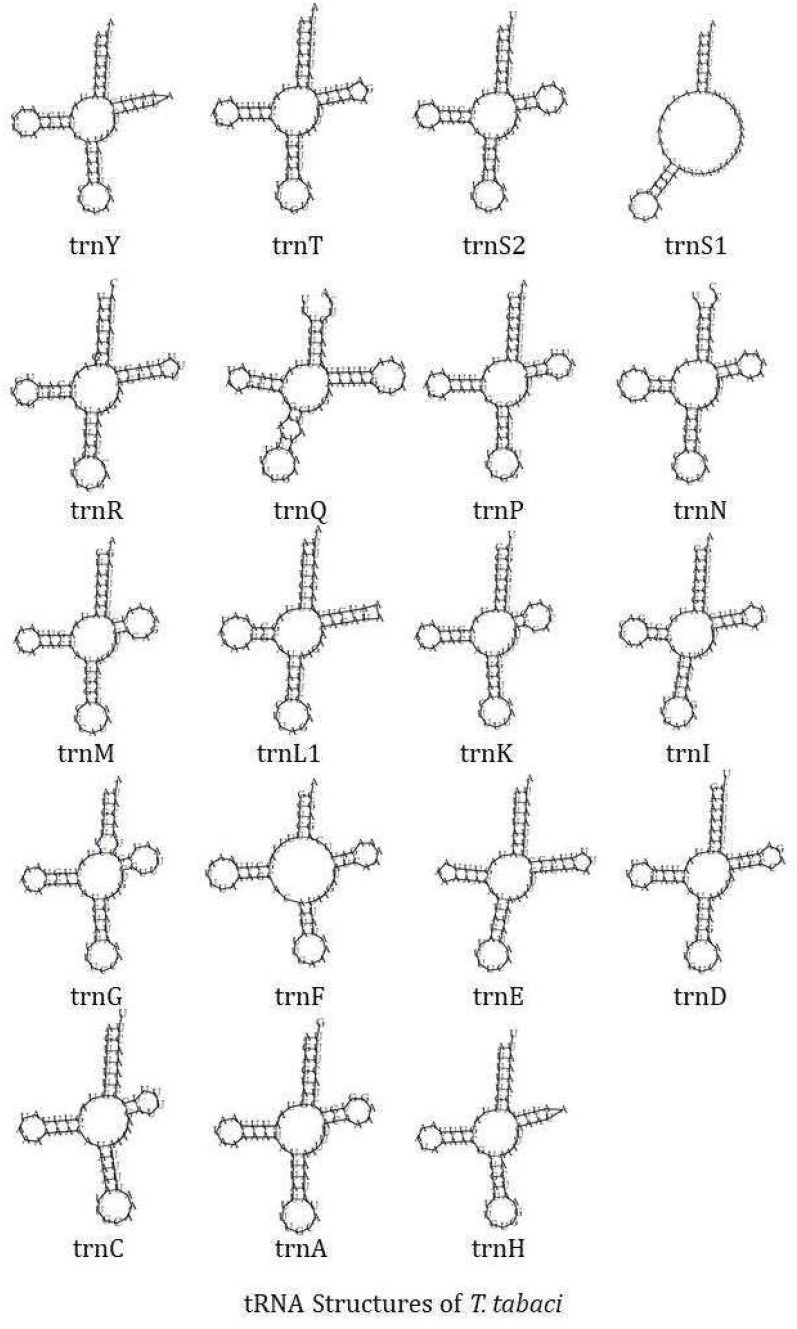
tRNA structures of the *T. tabaci*.

**Figure 4:**
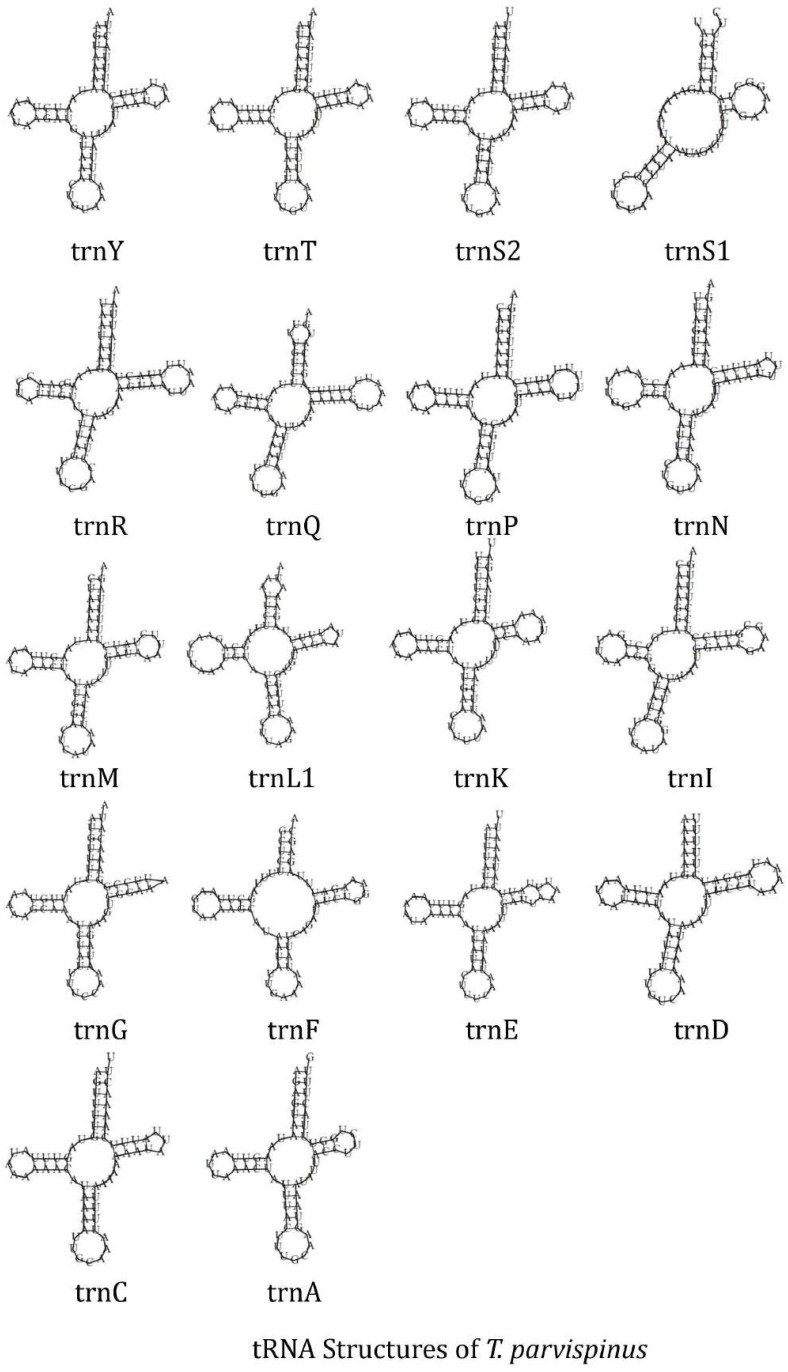
tRNA structures of the *T. parvispinus*.

The circular mitochondrial genome of *T. tabaci* contains 19 tRNA genes ranging in size from 57 to 68 bp. In the mitogenome, 13 of the tRNA genes are located on the H-strand (+), while the remaining six are located on the L-strand (–) (**Figure 1A** and **Table 1**). The 19 tRNA-coding genes of *T. tabaci* mitochondrial genome collectively comprised 1,200 bp, representing 7.85% of the entire mitogenome. In *T. parvispinus* mitogenome, 18 tRNA-coding genes ranging in size from 61 to 69 bp were detected. All of the tRNA-coding genes of *T. parvispinus* were located on the H-strand (+) with the exception of two genes encoding proline (Pro/P) and tyrosine (Tyr/Y) located on the L-strand (-) of mitogenome (**Figure 1B** and **Table 3**). These 18 tRNA-coding genes of *T. parvispinus* comprise a 1,170-bp region that covers 7.65% of the mitogenome. The characteristic clover leaf secondary structures of tRNA genes were identified in the mitogenomes of both thrips species, with the exception of trnS1, which lacks a dihydrouridine arm (**Figures 3**, **4**). Three genes encoding tRNAs for leucine (Leu/L2), tryptophan (Trp/W), and valine (Val/V) were lacking in the mitogenome of *T. tabaci*; however, in addition to these three genes, histidine (His/H) was missing in the *T. parvispinus* mitogenome.

The rRNA genes rrnL and rrnS collectively constitute 11.51% of the total mitogenome. The rrnS gene (607 bp long with 76.80% A+T content) was situated between the atp8 and tRNA-Phe genes. The rrnL gene (1,152 bp long with 79.30% A+T content) was located between the nad6 and cox1 genes of the *T. tabaci* mitogenome. However, in the *T. parvispinus* mitogenome, both the rRNA-coding genes rrnL (1148 bp) and rrnS (753 bp) were found on the H-strand (+) and covered 12.43% (1901 bp) of the whole mitochondrial genome.

### Phylogenetic analysis

**Figure 5:**
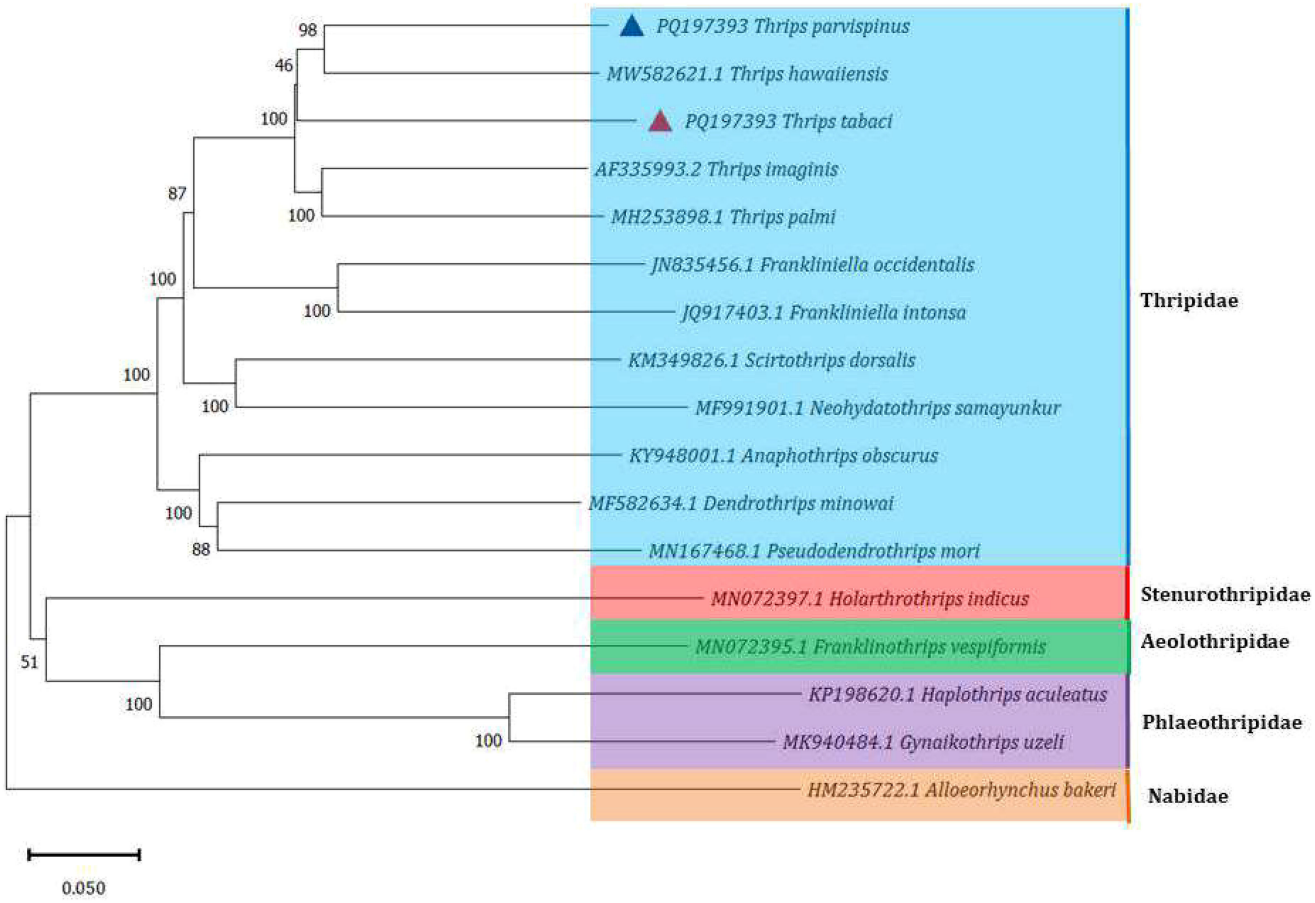
Phylogenetic analysis of 16 Thrips species, with the damsel bug, *Alloeorhynchus bakeri* as outgroup. (*T. tabaci* and *T. parvispinus* marked with red and blue triangles respectively are from current study).

Species within the same family, Thripidae, *T. palmi*, *T. imagines*, *T. hawaiiensis*, F*. intonsa*, *F. occidentalis*, *Dendrothrips minowai, Pseudodendrothrips mori, Neohydatothrips samayunkur, Scirtothrips dorsalis, Anaphothrips obscurus*, were grouped together with *T. tabaci* and *T. parvispinus*, The species from other thrips family such as Stenurothripidae, Aeolothripidae and Phlaeothripidae were grouped in another cluster. The *T. parvispinus* was closely related to *T. hawaiiensis* with 98 bootstrap value. However, *T. tabaci* was slightly outgrouped from *T. hawaiiensis.* The *F. intonsa*, and *F. occidentalis* were cluster separately from species for genus *Thrips* (**Figure 5**). The other cluster included *Holarthothrips indicus* of the Stenurothripidae family grouped with 51 Bootstrap value with *Franklinothrips vespiformis* of the Aeolothripidae family, *Gynaikothrips uzeli*, and *Haplothrips aculeatus* of the Phlaeothripidae family. The damsel bug, *Alloeorhynchus bakeri*, was used in the dataset as an outgroup species.

### Control, overlapping, and intergenic spacer regions

The *T. tabaci* and *T. parvispinus* mitochondrial genome contains one control in both species, each of which is 451 bp and 347 bp, respectively. In *T. tabaci*, it is located between the trnD and trnR genes; however, in *T. parvispinus*, it is situated between the trnE and trnP genes. The percentages of AT in the control *T. tabaci* and *T. parvispinus* regions were 62.7% and 76.7%, respectively. T-stretch, GAnT motif, ATnC motif, tandem repeats, and stem loops were found in the control region of the *T. tabaci* mitogenome. However, the T-stretch followed by the TATA box, GAnT motif, tandem repeats, and stem loop were found in the *T. parvispinus* mitogenome.

The *T. tabaci* mitochondrial genomes contain 22 intergenic spacer regions of length 1109 bp that vary in length from 2 to 292 bp. There were 13 major intergenic spacers >10 bp in length observed in the circular genome. The longest intergenic spacer regions (292 bp) were located between nad4 and nad4L genes. However 9 overlapping sequences were found in *T. tabaci* mitochondrial genome, ranging in size from 1 to 54 bp. Similar to *T. tabaci*, *T. parvispinus* mitochondrial genome contained 22 intergenic spacer regions of total 692 bp, varying in length from 1 to 101 bp. There were 14 major intergenic spacers >10 bp in length observed in the *T. parvispinus* mitogenome. The cytB genes were followed by the longest intergenic spacer region of 101 bp. The 7 overlapping sequences were present in *T. tabaci* mitochondrial genome, ranging from 1 to 35 bp in size. Amid the nad4 and nad4L genes of the *T. tabaci* mitogenome, there were 292 bp noncoding nucleotides; however, *T. parvispinus* revealed 47 bp.

## Discussion

The circular mitogenome of *T. tabaci* and *T. parvispinus* from the present study revealed 15,277 bp and 15,285 bp. respectively. A similar trend was observed in most of the thrips species reported earlier. For instance, *T. imagines* (15,407 bp) (21), *T. palmi* (15,333 bp) (26), *Frankliniella intonsa* (15,215 bp) (23), *Frankliniella occidentalis* (14,889 bp) (22), *Scriptothrips dorsalis* (15,343 bp) (24), and *Anaphothrips obscurus* (14,890 bp) (25).

The gene order among these thrips species showed variations, especially within the subfamily Thripinae. In *T. tabaci* and *T. parvispinus* the several tRNA genes from both *T. tabaci* and *T. parvispinus* have been translocated, showing the variation and arrangement of the gene. Similar variation in the order of the mitogenome genes of the all thrips species was observed. This variation can provide insights into evolutionary processes and mechanisms of genetic diversity. It also highlights the complexity of mitochondrial inheritance and their adaptation to various environments at the genetic level (47). These variations might affect their physiology, behavior, and ecological interactions, making them an interesting subject for evolutionary studies (48). Additionally, understanding these evolutionary patterns can help in pest control strategies, as some thrips species are significant agricultural pests.

The RSCU data analysis revealed that Lysine followed by phenylalanine, leucine, isoleucine, tyrosine, and serine, are the most frequently used amino acids, which is common in most of the thrips species (23, 25, 26). The characteristic clover leaf secondary structures of tRNA genes were identified in the mitogenomes of both thrips species, with the exception of trnS1, which lacks a dihydrouridine (DHU) arm, which is common in most insect species. The DHU arm in the trnS1 secondary structure was missing in marigold thrips (*Neohydatothrips samayunkur*) (49), green semilooper (*Chrysodeixis acutaI*) (50), and Indian dammer bee, *Tetragonula iridipennis* (51).

In both the thrips species, some tRNA-coding genes, namely Leucine (Leu/L2), Tryptophan (Trp/W), and Valine (Val/V) were missing in *T. tabaci*, and in addition to these three, Histidin (His/H) is missing in *T. parvispinus* mitogenome. The presence of duplicate copies of tRNA in some thrips species and missing tRNA genes in Gynaikothrips has been reported by Tyagi et al. (52). However in many insect species mitochondrialt tRNA genes reported as lost or missing, on manual annotation found to have unusual secondary structures and contain many nucleotide mismatches (53). In some of the insect species, the truncated tRNA was observed, which formed during their evolution (54). The truncation of tRNA genes poses problems in locating and annotating them due to a high level of nucleotide mismatches (55). In the absence of a well-paired acceptor stem, the 3’ end is not clearly defined. The region downstream from the anticodon stem is extremely variable in sequence and length (54).

The 7 overlapping sequences were present in the *T. tabaci* mitochondrial genome, ranging from 1 to 35 bp in size. Amid the nad4 and nad4L genes of the *T. tabaci* mitogenome, there were 292 bp noncoding nucleotides; however, *T. parvispinus* revealed 47 bp, as in *Aeolothrips xinjiangensis* (148 bp) (48). Most of the thrips mitogenomes exhibited overlaps of 1-21 bp (mostly around 7 bp) between the nad4 and nad4L. In insect mitogenomes, the nd4 to nd4L regions are transcribed into polycistronic mRNA with either overlaps or no intergenic spacers between them (56–58). However, such a long intergenic spacer might split this polycistronic mRNA into two monocistronic mRNAs (48).

The phylogenetic analysis revealed that the genera Thrips and Frankliniella are closely related, as they cluster together. This close relationship is attributed to their shared homology of paired ctenidia on abdominal segments V-VIII (59). *T. tabaci*, *T. palmi*, *F. accidentalis*, and *F. intonsa* are known vectors of topoviruses (47). These species cluster together, suggesting they share similar genomic characteristics.

As like the genomic characteristics, the biological similarities are also present in *T. tabaci* and *T. parvispinus*. Field emission scanning electron microscopy (FESEM) analysis of both thrips species identified similar types of sensilla, including sensilla basiconica (SBI, SBII, SBIII), sensilla chaetica (SChI, SChII), sensilla trichodea (ST), sensilla campaniformia (SCa), and sensilla cavity (SCav); however, variations in the length of these sensilla were observed between the two species. Also, some morphological characters have the variation in both the thrips species, such as antennae (seven-segmented with forked sensorium on third, and fourth segments), ctenidia (paired ctenidia were present in 5th–8th abdominal segments laterally), and pronotum (two pairs of posteroangular setae) (60).

Mitochondrial genome data have been widely used for phylogenetic, evolutionary studies, and population genetics in insects (61). Among eukaryotes, Thysanoptera, along with other minor paraenopteran insect orders, is regarded as a model for rapid mitochondrial genome evolution (24). In the Thysanoptera order, thrips display exceptional interspecific variation in mitogenomic structure, making them an ideal model for studying mitochondrial evolution (48). In the current study, the several tRNA genes from both *T. tabaci* and *T. parvispinus* have been translocated; however, the protein-coding genes and most of the tRNA genes have a similar arrangement, representing that these two species have a low rate of the rearrangement among them, which may be originating from the most recent common ancestors of these thrips species. Similar results were reported by Yan et al. (23) in flower thrips, *Frankliniella intonsa*, and other thrips species. Mitochondrial DNA plays a crucial regulatory role in insect adaptation to environmental changes, including insecticide resistance (62). Many insecticides target mitochondrial functions, such as oxidative phosphorylation. Mutations in mtDNA can lead to changes in these functions, resulting in resistance to the insecticides (63). Mutations in the mitochondrial-encoded Cytb gene have been implicated in resistance to the novel acaricide Bifenazate in *Tetranychus urticae* and *Panonychus citri* (64). Furthermore, a newly identified mutation in the Cytb gene of *T. urticae* has been associated with resistance to the miticide Acequinocyl (65). However, in the current study, no such mutation was detected in the mitochondrial genes. Also, no report was found in any thrips species to date detecting insecticidal resistance in the thrips species due to mutation in the mitochondrial gene.

The phylogenetic analysis includes species from different thrips families, such as Thripidae, Aeolothripidae, Phlaeothripidae, and Stenurothripidae, showing the phylogenetic diversity among these groups. The diversity within these groups may contribute to the complexity and lower bootstrap values in some parts of the tree. The thrips species clustered together such as *T. hawaiiensis* and *T. parvispinus*, with bootstrap values 98, have closely related species *T. tabaci* with lowest bootstrap value 46, suggesting lower confidence in their relationships. The thrips species in phylogenetic study other than the Thripidae family, *Holarthrothrips indicus* of Stenurothripidae family, and *Franklinothrips vespiformis* of Aeolothripidae, *Gynaikothrips uzeli* and *Haplothrips aculeatus* of the family Phlaeothripidae shown the lower boot strap values, 51. The lower bootstrap values of these species could be because of the low rate of genetic flow between these species (66).

## Conclusion

Complete mitochondrial genome sequences of *T. tabaci* and *T. parvispinus* provide critical insights into their genetic makeup and evolutionary relationships. The mitochondrial genomes of *T. tabaci* and *T. parvispinus* were found to be 15,277 bp and 15,285 bp in size, respectively, with similar gene organization, including 13 protein-coding genes, 2 rRNA genes, and 19 tRNA genes in *T. tabaci*, and 18 tRNA genes in *T. parvispinus*. The high AT content observed in both species reflects their typical insect mitochondrial genome characteristics, which are essential for understanding their genetic diversity and evolutionary adaptations. Phylogenetic analysis revealed the evolutionary positions of *T. tabaci* and *T. parvispinus* within the Thysanoptera order, highlighting the genetic makeup and taxonomy of these species with other thrips species. The findings emphasize the significance of understanding the genetic basis of onion thrips, which can aid in developing targeted pest management strategies to mitigate their impact on onion production. The detailed mitochondrial genome sequences and phylogenetic analyses presented in this study provide a valuable resource for further research on thrips biology, genetics, and pest management.

## Data Availability

The datasets presented in this study can be found in online repositories. The names of the repository/repositories and accession number(s) can be found in the article/supplementary material.
